# “You do need to do the interaction”: Mothers’ perceptions of responsive parenting following a home‐based parenting intervention

**DOI:** 10.1002/imhj.22037

**Published:** 2023-03-03

**Authors:** Sophie Stucley Morris, Anna Price, Vicki McKenzie, Lynn Kemp

**Affiliations:** ^1^ Melbourne Graduate School of Education University of Melbourne Parkville Victoria Australia; ^2^ Centre for Community Child Health The Royal Children's Hospital Parkville Victoria Australia; ^3^ Population Health Murdoch Children's Research Institute Parkville Victoria Australia; ^4^ Department of Paediatrics University of Melbourne Parkville Victoria Australia; ^5^ Ingham Institute Western Sydney University Penrith South New South Wales Australia

**Keywords:** attachment, home visits, parent‐child relationship, parenting, qualitative, responsivity, capacidad de respuesta, crianza, afectividad, visitas a casa, cualitativo, Réactivité, parentage, attachement, visites à domicile, qualitatif, Responsivität, Elternschaft, Bindung, Hausbesuche, qualitativ, 応答性、 養育、 愛着、 家庭訪問、 質的, 关键词:响应, 育儿, 依恋, 家访, 定性, **الكلمات المفتاحية**: الاستجابة ، التربية ، التعلق ، الزيارات المنزلية ، الدراسة الوصفية

## Abstract

Responsive parenting (also known as responsivity) is a dynamic and bidirectional exchange between the parent‐child dyad and associated with a child's social and cognitive development. Optimal interactions require a sensitivity and understanding of a child's cues, responsiveness to the child's need, and a modification of the parent's behavior to meet this need. This qualitative study explored the impact of a home visiting program on mothers’ perceptions of their responsivity to their children. This study is part of a larger body of research known as *right@home*, an Australian nurse home visiting program promoting children's learning and development. Preventative programs such as *right@home* prioritize population groups experiencing socioeconomic and psychosocial adversity. They provide opportunities to promote children's development through the enhancement of parenting skills and an increase in responsive parenting. Semi‐structured interviews were conducted with 12 mothers, providing insight into their perceptions of responsive parenting. Four themes were extracted from the data using inductive thematic analysis. These indicated that: (1) mothers’ perceived preparation for parenting, (2) recognition of mother and child needs, (3) response to mother and child needs, and (4) motivation to parent with responsiveness, were considered important. This research highlights the importance of interventions that focus on the parent‐child relationship in increasing mother's parenting capabilities and promoting responsive parenting.

## Resumen

La crianza con sensibilidad (también conocida como crianza con capacidad de respuesta) es un dinámico intercambio en ambas direcciones entre la díada progenitor‐niño, asociada con el desarrollo social y cognitivo del niño. Las interacciones óptimas requieren de una sensibilidad y comprensión de las señales del niño, capacidad de respuesta a la necesidad del niño, y de una modificación del comportamiento del progenitor para cumplir con esta necesidad. Este estudio cualitativo exploró el impacto de un programa de visita a casa sobre las percepciones de las madres acerca de su capacidad de respuesta a sus niños. Este estudio es parte de un mayor cuerpo investigativo conocido como right@home (justo en casa), un programa australiano de visita a casa por parte de enfermeras que promueve el aprendizaje y desarrollo de los niños. Los programas preventivos tales como right@home dan prioridad a grupos de población que experimentan adversidad socioeconómica y sicosocial. Ellos ofrecen oportunidades para promover el desarrollo de los niños por medio del mejoramiento de las habilidades de crianza y un incremento de la sensibilidad en la crianza. Se llevaron a cabo entrevistas semiestructuradas con 12 madres, lo cual aportó ideas sobre sus percepciones de la crianza con sensibilidad. Se extrajeron cuatro temas de la información usando análisis temáticos inductivos. Estos indicaron que se consideraban importantes (1) el cómo las madres percibían su preparación para la crianza, (2) el reconocimiento de las necesidades de la madre y del niño, (3) la respuesta a las necesidades de la madre y del niño, y (4) la motivación a criar con sensibilidad. Esta investigación subraya la importancia de intervenciones que se enfoquen en la relación progenitor‐niño para aumentar las capacidades de crianza de la madre y promover una crianza con sensibilidad.

## Résumé

Le parentage réactif (aussi connu comme réactivité) est un échange dynamique et bidirectionnel entre la dyade parent‐enfant, et lié au développement social et cognitive d'un enfant. Les interactions optimales demandent une sensibilité et une compréhension des signaux de l'enfant, la réactivité au besoin de l'enfant, et une modification du comportement du parent pour répondre à ce besoin. Cette étude qualitative a exploré l'impact d'un programme de visite à domicile sur les perceptions des mères de leur réactivité à leurs enfants.

Cette étude fait partie de recherches plus étendues connues sous le nom de de right@home, un programme de visite infirmières à domicile australien promouvant l'apprentissage et le développement des enfants. Les programmes de prévention tels que right@home privilégient les groupes de population marqués par l'adversité socioéconomique et psychosociale. Ils offrent des opportunités de promouvoir le développement des enfants au travers du renforcement des capacités de parentage et une augmentation du parentage réactif.

Des entretiens semi‐structurés ont été faits avec 12 mères, offrant un aperçu de leurs perceptions du parentage réactif. Quatre thèmes ont été extraits des données en utilisant une analyse thématique inductive. Ces dernières ont indiqué que (1) la préparation perçue des mères pour le parentage, (2) la reconnaissance des besoins de la mère et de l'enfant, (3) la réponse aux besoins de la mère et de l'enfant, et (4) la motivation du parent avec la réaction ont toutes été considérées comme importantes. Ces recherches mettent en lumière l'importance d'interventions qui se concentrent sur la relation parent‐enfant en augmentant les capacités de parentage des mères et en promouvant le parentage réactif.

## Zusammenfassung

„Sie müssen die Interaktion durchführen": Wie Mütter responsive Elternschaft nach einer häuslichen Erziehungsintervention wahrnehmen

Responsive Elternschaft (auch bekannt als Responsivität) ist ein dynamischer und bidirektionaler Austausch zwischen der Eltern‐Kind‐Dyade, der mit der sozialen und kognitiven Entwicklung des Kindes im Zusammenhang steht. Optimale Interaktionen erfordern Sensibilität und Verständnis für die Signale des Kindes, das Eingehen auf die Bedürfnisse des Kindes und eine Modifizierung des elterlichen Verhaltens, um diese Bedürfnisse zu erfüllen. In dieser qualitativen Studie wurde untersucht, wie sich ein Hausbesuchsprogramm auf die Wahrnehmung von Müttern hinsichtlich ihrer Responsivität gegenüber ihren Kindern auswirkt.

Diese Studie ist Teil eines größeren Forschungsprojekt namens right@home, ein australisches Programm, das durch Hausbesuche von Pflegekräften das Lernen und die Entwicklung von Kindern fördert. Präventive Programme wie right@home richten sich vorrangig an Bevölkerungsgruppen, die von sozioökonomischen und psychosozialen Widrigkeiten betroffen sind. Sie bieten die Möglichkeit, durch die Verbesserung elterlicher Fähigkeiten und die Steigerung elterlicher Responsivität kindliche Entwicklung zu fördern.

Halbstrukturierte Interviews wurden mit 12 Müttern geführt, die einen Einblick in ihre Wahrnehmung von responsiver Elternschaft gaben. Mittels induktiver thematischer Analyse wurden vier Themen aus den Daten extrahiert. Wichtige Themen waren (1) die von den Müttern wahrgenommene Vorbereitung auf die Elternschaft, (2) das Erkennen der Bedürfnisse von Mutter und Kind, (3) das Eingehen auf die Bedürfnisse von Mutter und Kind und (4) die Motivation, als Elternteil responsiv zu sein. Diese Untersuchung unterstreicht die Bedeutung von Interventionen, die sich auf die Eltern‐Kind‐Beziehung konzentrieren, um die elterlichen Fähigkeiten der Mutter zu verbessern und eine responsive Elternschaft zu fördern.

## 抄録

“あなたにこそ相互交流が必要です”　在宅育児介入後の応答的な育児に対する母親の認識

応答性のよい子育て (応答性) は、親‐子間のダイナミックで双方向的な交流であり、子どもの社会的・認知的発達に関連する。最適な相互交流は、子どもの合図に対する感受性と理解、子どものニーズに対する応答性、そしてそのニーズにふさわしいように親の行動の修正を必要とする。この質的研究は、家庭訪問プログラムが、母親の子どもへの応答性の認知に対して与える影響を調査した。

この研究は、子どもの学習と発達を促進するオーストラリアの看護師による家庭訪問プログラムであるright@homeとして知られる、より大規模な研究群の一部である。right@homeのような予防的プログラムは、社会経済的・心理社会的な逆境を経験している集団を優先する。子育てスキルの強化と応答的な子育てを増やすことで、子どもの発達を促進する機会を提供する。

12人の母親を対象に半構造化面接を実施し、応答的な子育てに対する母親の認識について洞察を得た。帰納的主題分析により、データから4つのテーマが抽出された。その結果、(1)母親の認識した子育てのための準備、(2)母親と子どものニーズの認識、(3)母親と子どものニーズへの反応、(4)応答性ある子育てへの動機づけ、が重要であると考えられた。この研究は、母親の子育て能力を高め、応答性のある子育てを促進するためには、親‐子関係に焦点を当てた介入が重要であることを明らかにした。

## 摘要

摘 要:响应式育儿 (也称为响应性) 是父母与孩子之间动态和双向的交流, 与孩子的社会和认知发展有关。最佳的互动需要对孩子的暗示保持敏感和理解, 对孩子的需求做出反应, 并改进父母的行为以满足这种需求。这项定性研究探讨了家访计划对母亲响应孩子的影响。

本研究是一个名为right@home的大型研究项目的一部分, 该项目是一个澳大利亚护士家访计划, 旨在促进儿童的学习和发展。诸如right@home这样的预防性计划, 优先考虑经历社会经济和社会心理困境的人群。它们通过提高育儿技能和增加响应式育儿, 为促进儿童的发展提供了机会。

研究人员对12名母亲进行了半结构化访谈, 深入了解了她们对响应式育儿的看法。采用归纳主题分析的方法从数据中提取出四个主题。主题 (1) 母亲对育儿的感知准备, (2) 对母子需求的认知, (3) 对母子需求的响应, (4) 对父母响应的动机, 被认为是重要的。本研究强调了以亲子关系为重点的干预措施在提高母亲育儿能力和促进响应式育儿方面的重要性。

## ملخص

“ **أنت بحاجة إلى القيام بالتفاعل”: تصورات الأمهات عن التربية المستجيبة بعد تدخل رعاية والدية في المنزل **

التربية المستجيبة هي تبادل ديناميكي وثنائي الاتجاه بين الوالد والطفل ، وترتبط بالتطور الاجتماعي والمعرفي للطفل. تتطلب التفاعلات المثلى حساسية وفهم إشارات الطفل والاستجابة لاحتياجات الطفل وتعديل سلوك الوالدين لتلبية هذه الحاجة. استكشفت هذه الدراسة الوصفية تأثير برنامج الزيارة المنزلية على تصورات الأمهات لاستجابتهن لأطفالهن.تعد هذه الدراسة جزءًا من مجموعة أكبر من الأبحاث تُعرف باسمright @ home ، وهو برنامج زيارة منزلية للممرضات الأسترالية يعزز تعلم الأطفال ونموهم. تعطي مثل هذه البرامج الوقائية الأولوية للفئات السكانية التي تعاني من صعوبات اجتماعية اقتصادية ونفسية اجتماعية. كما توفر فرصًا لتعزيز نمو الأطفال من خلال تعزيز مهارات الأبوة والأمومة وزيادة استجابة الأبوة والأمومة. تم إجراء مقابلات شبه منظمة مع 12 من الأمهات لاستكشاف لتصوراتهن عن التربية المستجيبة. تم استخراج أربعة محاور من البيانات باستخدام التحليل المواضيعي الاستقرائي. أشارت هذه النتائج إلى أهمية (1) استعداد الأمهات لتربية الأبناء ، (2) الاعتراف باحتياجات الأم والطفل ، (3) الاستجابة لاحتياجات الأم والطفل ، (4) الدافعية للوالدين ذوي الاستجابة. يسلط هذا البحث الضوء على أهمية التدخلات التي تركز على العلاقة بين الوالدين والطفل في زيادة قدرات التربية لدى الأم وتعزيز التربية المستجيبة.

## 1 INTRODUCTION

The earliest years of life are critical for the development of language, cognitive capacity, and socio‐emotional abilities; these, in turn, influence the trajectory of learning, school achievement, and later life satisfaction (Britto et al., 2016; Pavalko & Caputo, 2013; Raby et al., 2015; Van Der Voort et al., 2014). The parent‐child relationship has long been recognized as a significant factor in promoting positive childhood development and mental health.

Interventions targeting the relationship between parents and their infants often focus on levels of attachment, or a related construct, responsive parenting. Parental responsiveness (also known as responsivity) is a process of interaction that determines the quality of the parent‐child attachment (Moore et al., 2013). Whilst attachment refers to the internal state of affectional bonding between parent and child, responsivity is an observable behavior that is key in promoting secure attachments (Black & Aboud, 2011). Guralnick (2006) reviewed the literature on family interaction patterns and their influence on child social and cognitive development. The authors describing parental responsivity as a “sensitive responsiveness” to the child, and highly influential for child development. Responsive parenting can be described using the framework of attachment theory, as the ability of the parent to actively connect with their child, and the capacity to read and respond to the child's needs (Landry et al., 2008). To be responsive, the parent must respond to the child in a timely, predictable, and appropriate manner, as well as communicate to the child that their interests, desires, and interactions have importance and influence (Eshel et al., 2006; Guralnick, 2006). Responsive parenting is thus a dynamic and bidirectional exchange between the parent‐child dyad, with links to a child's social and cognitive development (Black & Aboud, 2011).

Socioeconomic and psychosocial adversity (such as financial hardship, poor mental health, low social support) can disrupt the opportunities and capacity for responsive parenting (Moore et al., 2013). Parenting programs are often utilized as an intervention for families experiencing adversity. They support parents to build skills in responding to infant/child behavior and reading cues, and promote parental self‐perception (Britto et al., 2016; Korom & Dozier, 2021). Interventions aimed at facilitating the parent‐child relationship often focus on increasing responsive parenting rather than attachment, as responsive parenting is outwardly observable and easier to address (Juffer et al., 2017; Moore et al., 2013). One of the most widely‐implemented parenting programs comprises intensive and sustained home visits by health professionals in the antenatal and postnatal period (Goldfeld et al., 2018; Kemp et al., 2008; Paton et al., 2013). In Australia, this is generally by child and family health nurses, who deliver primary healthcare for families with children from birth to school entry, within a universal health system (Goldfeld et al., 2018; Kemp et al., 2008; Paton et al., 2013). Home visiting programs generally aim to ameliorate preventable risk factors, including insecure attachment, by teaching strategies to assist with child and family health, increasing responsive parenting capacity, and developing parenting confidence and efficacy (Kemp et al., 2019; Olds et al., 2002; Paton et al., 2013). Other programs such as *right@home* (the focus of this study) include specific intervention strategies to enhance responsivity such as video feedback (Juffer et al., 2017; Newton et al., 2022).

**RELEVANCE**

Responsive parenting is a dynamic and bidirectional exchange between the parent‐child dyad and associated with a child's social and cognitive development. Responsive parenting can be harder when parents are faced with socioeconomic and psychosocial adversities. This study demonstrates how a systematic and sustained intervention with high intensity of supports can benefit mothers’ understanding of and ability to provide responsive parenting.

**Key Findings**

1. Responsive parenting was a product of the dynamic interaction between a woman's natural propensities and her learnt experience, which resulted in motivated, practiced, and reinforced behaviors.
2. Developing and practicing the skills for responsive parenting requires commitment from the mother and health practitioner (in this study, highly skilled child and family health nurses), and social and professional supports.
3. Programs such as nurse home visiting are of value and should focus on behavioral skills or the actions needed to parent (rather than vague or unclear information); promoting the parent‐child relationship; and validating the mother's experience.

Whilst there is substantial literature exploring the benefits of increasing parental responsivity via a sustained home‐based nursing program, there is less understanding of the process by which this change occurs. Thus, the current study sought to explore the mother's perspective of responsivity and how it is developed in a home visiting program. We aimed to develop a model that compared how the underlying program logic of a sustained home visiting intervention, *right@home* (the message delivered), matched the experience of the participant (the message received) (Kemp et al., 2013). To address these aims, and based on theories of responsive parenting, this three‐part research question was posed:

How has participation in the *right@home* intervention influenced the parent's:

1. Abilities to actively connect with their child?
2. Capacity to read their child's needs?
3. Perception of how confident they feel in their parenting ability?

## 2 METHOD

### 2.1 Design

A qualitative exploratory research design was employed utilizing a constructionist approach to ensure the participants’ physical, psychological, social, and cultural contexts were represented in the data (Berg, 2007). Participants’ perceptions of parental responsivity were explored, as well as elucidation about how the intervention had an impact. This approach was based on phenomenology, aiming to elicit the individual's perspective and own understanding of their experience, and how they theorize about their own behavior (Berg, 2007).

### 2.2 Recruitment

This qualitative study was nested within the *right@home* randomized control trial (RCT) of sustained nurse home visiting (details described in the protocol, Goldfeld et al., 2017). Women were approached for the larger RCT while pregnant and attending antenatal clinics of ten birthing hospitals across the states of Victoria and Tasmania in Australia. Those who fulfilled the eligibility criteria, expressed interest in participating, and provided informed consent were enrolled into the trial. Participants for the current study were recruited from the sample of mothers enrolled in the *right@home* intervention. Trained *right@home* research assistants sought expressions of interest from all participants of the larger study via phone, with the final participants selected by the lead author of this nested study.

To be included in the current study, participants must have been 6 months post completion of the *right@home* intervention at child‐age 2‐years, as well as be available and agreeable for a phone interview with the lead author. This timeframe was chosen to allow participants time to have reflected on their experience of the program and an opportunity to have implemented program teachings. Participants were selected using both purposeful stratification sampling and theoretical sampling. This sampling strategy allowed participants to be selected from a sample of 72 women who received the intervention and who had consented to be interviewed. Participant selection was based on demographic factors that were hypothesized from the previous literature to be associated with responsivity, allowing for information‐rich cases to be studied in depth and in an iterative manner (Palinkas et al., 2015; Paton et al., 2013). Pre‐specified stratification factors included the mothers’ age, employment status, education status, marital status, multiparous status, and cultural background. Data analysis was undertaken concurrently with recruitment and interviews. This made it possible to confirm the final number of participants needed so that the information analyzed was adequate to answer the research questions. It also made it possible to develop a conceptual understanding of responsive parenting; in other words, when the analysis had adequate information power (Braun & Clarke, 2021). A total of 12 participants were interviewed and included in data analysis (see Table 1).

**TABLE 1:** General participant information.

Pseudonym	Age at birth of child	First child	Risk factors at antenatal trial recruitment screening	Number of risk factors^a^
Penny	22	No	Age < 23 Poor/fair/good health^b^	2
Bella	23	Yes	Poor/fair/good health Illness	2
Mei	35	Yes	Not living with another adult Poor/fair/good health	2
Angela	37	No	Poor/fair/good health Significant stress	2
Laura	18	Yes	Age < 23 Poor/fair/good health Schooling < year 12	3
Tara	37	No	Poor/fair/good health Illness	2
Jennifer	20	No	Age < 23 Poor/fair/good health No household income	3
Ashley	32	No	Schooling < year 12 No household income Never had a job	3
Vanessa	44	Yes	Illness Schooling < year 12	2
Michelle	26	No	Poor/fair/good health Smoker Schooling < year 12	3
Tiffany	26	Yes	Illness Significant stress	2
Melissa	28	No	Not living with another adult Schooling < year 12 No household income	3

^a^A minimum of two screened risks (of ten) were required for participation in the *right@home* trial (Goldfeld et al., 2017).

^b^Poor/fair/good health versus very good/excellent health.

### 2.3 Measures

A semi‐structured interview schedule was designed to elicit complex narratives of mothers’ experiences of parenting, the *right@home* intervention, and other supports (see Box 1). Minor modifications to questions occurred during the interviews to ensure that questions were appropriate to the participant's needs and effective in eliciting information. These included allowing a recursive style of questioning, enabling minor changes to wording, and additional prompts for participants to tell their story (Minichiello et al., 1995).

**Box 1 Interview schedule with prompts**

Interview questions

1. Thinking back to when you first came home from hospital with <CHILD> and the first 8 weeks what were some of the things you found useful in helping your parenting?
2. How about after you had some time to adjust and get to know <CHILD>. How was the first year?
3. And what about until <CHILD> turned two?

(PROMPT: What did you find difficult or useful?—Parenting skills. How would you describe the change in yourself as a parent from 2 years ago to now? What sort of things did you find yourself doing differently?)

1. What did you do when you wanted some advice or help with <CHILD>?

(PROMPT: Where did you go for help? Who did you speak to?)

1. From what you have learnt what advice would you give new parents?
2. What was it like when the nurse visited?
3. In what ways did the visits help you?
4. In what ways were they unhelpful?

(PROMPT: Older/younger children, first time parenting, difference in what you imagine you'd do—what do you think might have happened if the nurse hadn't have visited)

1. What sort of differences did the visits make?
2. Did it make a difference in how you helped or parented your child?
3. Did it make a difference in how you think of yourself as a parent?
4. Did it change your relationship with your child?

(PROMPT: Did you feel you could better understand your child or respond to their needs?)

1. How did you get on with the nurse?
2. How did you feel when the nurse visited?
3. Did this change over the 2 years?
4. What advice could you give new mothers about having a nurse come visit?

(PROMPT: What would you have liked to have heard before you had a home visit?)

1. What do you think should happen with home visits?
2. For example, should they be for everyone, just people with difficulties, more often, just one off, longer, shorter, etc.
3. What would you change about the home visiting program?
4. How do you think this could help?
5. What would you have done without the intervention? (can be integrated throughout all questions)

(PROMPT: Where do you think you would have gone for help?)

*(If necessary, ask: And could you tell me who currently lives with <CHILD> and yourself?)*

For mothers with other children (multiparous) mothers only

1. How did the visits change how you parented your older child/children?
2. How did the visits change how you parented your younger child/children?

#### 2.3.1 Procedure

Details of the participants to be contacted for interview were given to the lead author, who conducted all interviews. With the participant's permission, interviews were recorded. Participants had the opportunity to ask any questions before and after the interview. The duration of the twelve interviews ranged from 15 to 39 min (*M* = 25.41, *SD* = 7.14).

### 2.4 Data analysis

An inductive thematic analysis was conducted of the data using recommendations from Braun & Clarke (2018). Thematic analysis is a qualitative methodology used to identify, analyze, and report patterns within and across data sets, with findings developed from the analytic processes following coding based on the close reading and interpretation of the data rather than pre‐determined theory (Braun & Clarke, 2018; Thomas, 2006). The inductive process of analysis allows for identified themes to be strongly linked to the data rather than the theoretical and epistemological preconceptions of the researcher, minimizing bias but not eliminating it (Braun & Clarke, 2018).

Braun & Clarke (2018) propose a six‐step model of thematic analysis: familiarity with data; transcription; generation of codes; search for themes; review, define and name themes; and report interpretive analysis with examples. Initial familiarity with the data was established through the lead author transcribing verbatim each interview. The lead author then re‐listened to the interview while reading the transcript to ensure accuracy and data validation, noting any initial ideas, thoughts, or possible codes.

Raw transcripts were loaded into the qualitative data analysis program NVIVO. Initial coding of one interview occurred with three members of the research team, to ensure interpretive and evaluative rigor (Morse, 2015). Analysis occurred alongside interviews and transcription and to enable the lead author to be aware of when saturation had been reached, and continue familiarity with the data. NVIVO allows for codes to be generated and assigned to data that is relevant to the research questions. These codes are viewable to the researcher on the right‐hand side of the transcription, allowing the researcher to engage in an active interpretive process on each re‐read of the transcript. Transcripts were re‐read until the lead author was satisfied that no new relevant data was able to be extracted from the transcript.

Patterns were identified in the data, and codes were analyzed and interpreted into overarching themes. Visual representations of possible links within and between interviews and themes were identified by the lead author after each transcription, being subsequently refined into an overarching model of parental responsivity.

### 2.5 Researcher reflexivity

The study team represent a range of disciplinary backgrounds including psychology, child and family health nursing, epidemiology, implementation, and evaluation. In addition, the senior author had considerable experience in interventions to improve responsivity, which may have influenced the expectations of behaviors to be observed in the participants. To minimize such influences, the initial analysis was conducted by the first author who was not engaged in any aspects of the *right@home* trial or intervention.

### 2.6 Ethics

The Royal Children's Hospital Human Research Ethics Committee approved *right@home* (HREC 32296), Australia.

## 3 RESULTS

Overall, the *right@home* intervention was viewed by the mothers as helpful in supporting their parent‐child relationship. The women were able to talk about their experience of the home visiting program over the previous 2 years, and provide insights into their parenting. The inductive thematic analysis using the interview data elucidated the construct of responsive parenting and how the intervention assisted in its positive expression.

Four main themes were extracted from the data, including three themes focusing on mothers’ perceptions of responsive parenting: (1) preparation for parenting, (2) recognition of needs, (3) responding to needs, and one theme about what assists responsive parenting, and (4) motivators. Table 2 outlines these themes and their sub‐themes, which are described in detail below. The themes involve some degree of overlap as the concepts explored are not mutually exclusive.

**TABLE 2:** Mothers’ perceptions of responsive parenting.

Core theme	Sub‐theme
1. Preparation for parenting	1.1. Drawing on past experience 1.2. Seeking advice 1.3. Practical set‐up 1.4. Managing expectations
2. Recognition of needs	2.1. Recognition of child's needs 2.1.1. Understanding the difference between children 2.1.2. Child's health and wellbeing 2.2. Recognition of parent's needs 2.2.1. Need to be supported 2.2.2. Need for self‐care, health, and wellbeing
3. Responding to needs	3.1. Modification of behavior 3.2. Open to change and flexibility 3.3. Acting in a child centric manner 3.4. Confidence in parenting ability
4. Motivators	4.1. Child outcomes 4.2. Nurse influence 4.3. Support systems

### 3.1 Theme 1. Preparation for parenting

All 12 mothers spoke about their preparation for parenting. This theme consists of a number of interrelated sub‐themes: drawing on past experiences, seeking advice, practical set‐up, and managing expectations. Mothers identified the factors they believe they need to succeed at parenting.

#### 3.1.1 Drawing on past experiences

Mothers spoke of the previous contact they have had with infants, whether it be children they have had prior to the intervention (*n* = 7), or experiences of being around young children or babies. “Being the second time around I guess it was easier because I knew what to expect.” (Angela).

A second mother commented that her experience of parenting her first two children was vastly different to her third and fourth, as there had been a gap of 8 years. She needed to re‐learn some aspects of parenting that had significantly changed to reflect current safety guidelines. She was happy to have help from the nurse in explaining current practice. “Some things have changed that I was unaware of, so it was good to know.” (Melissa).

Another participant reflected that as she had not had any previous experiences with children, she felt she needed to educate herself on the practicalities involved and seek advice from professionals and family. She observed that despite her preparation and research, her experience of early infancy was still challenging. She benefited from practical assistance learning the fundamentals of parenting (such as changing a nappy), as well as understanding the difficulty of translating information into action. “Learning all those basic things because you can read as much as you like, but once you get hands on it's a completely different thing. You have to adapt the way that you put that theory into practice.” (Vanessa).

#### 3.1.2 Seeking advice

All mothers interviewed mentioned the value of seeking advice from multiple sources. The mothers talked to the people available to them to develop an understanding of the range of behaviors to expect in their infants. This also gave parents information about what they could expect the stages of parenting to look like. Further, parents sought advice from “experienced” parents, acknowledging that capacity to parent increases with experience. “So being able to talk to other parents and say, ‘Is this normal?’ Or ‘have you got any advice for us?’ We tried to get advice from people who had been there before.” (Penny).

In addition to seeking advice from experienced parents, the mothers identified helpful sources including family, friends, and professional services to which they had access. One mother commented that she preferred to access advice from professionals whose advice was underpinned by evidence. “I like more of a bit of knowledge with probably a bit of study behind it, a bit of research behind it rather than just this worked for my baby.” (Bella).

#### 3.1.3 Practical set‐up

Practical set‐up involves identifying the physical requirements that assist in parenting and an appreciation of the need to change the home environment to adjust to having a new child. A number of mothers identified that having a newborn child would be a large time commitment. One mother spoke of how she anticipated having less time for other activities after the birth of her second child. This allowed her to plan for the time commitment necessary to care for her new infant as well as her existing family. “I made lots of meals to put in the freezer and made lots of goodie bags and things for my toddler.” (Penny).

Many mothers (*n* = 6) commented on having a family member, often their mother or mother‐in‐law, come and stay in the house after the birth of the child. This allowed for the mother to give the individual attention that the new baby needs whilst the additional carer focused on the practicalities of running the household. One mother spoke about the practical help offered by her mother‐in‐law after the birth of her child. Her mother‐in‐law came from interstate to stay for a week, offering assistance by cooking, cleaning, and looking after the three older children, as well as helping to care for the new baby. She trusted the help offered as the mother‐in‐law “had eight children of her own, so she kinda knows.” (Ashley). Another participant valued the support shown by her own mother moving in to live with her and sharing the burden of night‐time care. This began when the child was a newborn, and still continues. “My mum will take over and look after him in the morning, so that I can catch up or to have a little bit of sleep.” (Mei).

A separate component of practical set‐up was identifying the financial costs associated with a new child. Mothers who stayed home after the birth of the child wanted to plan for the loss of their income (*n* = 4). They recognized that there was benefit in organizing their finances early as this allowed a focus on the child rather than worrying about their financial situation. Even with this recognition of lost income and preparation, staying home and parenting a young child was still challenging. “We planned for her. We had enough savings and we had everything bought. Just the lack of sleep. I think that really threw us.” (Bella).

#### 3.1.4 Managing expectations

The mothers spoke of the importance of having realistic expectations of their capacity to provide everything they felt their child needed. Some mothers (*n* = 6) reflected on the importance of providing themselves with small manageable goals that were achievable given the constraints of parenting. “[I] was telling myself, ‘If I get to have a shower each day, that's a good thing.’ Small goals.” (Penny). Others (*n* = 5) reflected on their own experience of early parenting, admitting that they had unrealistic expectations of themselves. One mother felt that as she was not working and was at home, she would have enough time to make everything “perfect.” Thinking back, she recognized that this was unmanageable and that she was still learning the skills of balancing motherhood with other life demands. “It just doesn't work out that way. You're lucky to feed yourself and you try, but won't do everything right for the child, but the fact is you're still learning as well.” (Tara).

Another mother echoed the need to impose realistic expectations on what you can achieve. She felt that it was important to recognize that it was the unrealistic expectation rather than your performance as a parent that caused problems for new mothers. “Just because you can't do it doesn't mean you're a failure especially if it's your first child. I've got four and I'm still learning.” (Melissa).

### 3.2 Theme 2. Recognition of needs

All participants spoke about the theme of recognizing needs. This consists of two sub‐themes, recognizing the needs of the child, and recognizing the needs of the mother/self. Within this, mothers identified further aspects. There were two components to recognizing needs of the child: understanding the difference between children, and the child's health and wellbeing. There were two components in the sub‐theme of recognizing needs of parents: the need to be supported, the need for self‐care, health, and wellbeing.

#### 3.2.1 Recognition of the child's needs

Parents recognized their child's needs as an important factor in responsive parenting.

##### 3.2.1.1 Understanding the difference between children

Ten mothers articulated that their child is individual in their needs, and requires differential treatment to their other children. “What works for one won't necessarily work for the other.” (Penny).

Eight mothers spoke of how getting to know their child enabled them to be more responsive to their needs and emphasized the importance of making sense of their child's signals. “You get to know them. I guess you've got to work them out … Listen to your baby. Take your cues from them, because they know what they want and you've just got to read them and know them.” (Ashley).

Mothers spoke of the nuances in their own children that revealed the differences between siblings. One mother explained how her other children had given very different cues. Once the mother was able to recognize the differences, she could give a more sensitive and individually tailored response that was appropriate to that particular child. “She still lets you know in her own way but you've just got to watch for the signs of what she wants.” (Tara).

##### 3.2.1.2 Child's health and wellbeing

Mothers (*n* = 11) were consistent in emphasizing the need to understand the child's autonomy and individuality in order to support their wellbeing. They further expressed their capacity to better identify their child's health and wellbeing. A large range of factors were identified as contributing to the child's wellbeing, with the majority of mothers (*n* = 9) emphasizing that “the child needs a parent.”

There was recognition that the parent was capable of distinguishing the individual health and wellbeing needs of their child, and at times even better than professionals. “I went to a different GP and I said, ‘I know there's something wrong.’ She said, ‘Yeah. I'd definitely listen to a parent's gut feeling first.’ That got him diagnosed with a really dangerous gut issue that he had.” (Penny).

Some mothers (*n* = 8) attributed the increased time they spend with their child as giving them greater insight into their child's needs. “All the time that you stay with him, you kind of understand a little bit better than others.” (Mei).

#### 3.2.2 Recognition of the parent's needs

In addition to understanding the importance of reading their child's cues, all mothers talked of the importance of supporting their own needs.

##### 3.2.2.1 Need to be supported

All mothers acknowledged that they needed assistance to be able to parent successfully. This assistance was often in the form of asking for help, and in feeling that it was valid to seek advice. It was often in reflecting on their past behavior that mothers identified that having outside help could be of assistance. All mothers commented on how they felt supported by the *right@home* nurses. The nurses were able to offer support to mothers worried about their child's behavior or development, as they had the experience and knowledge to reassure parents that their child behavior, and the parent's responses, were normal. “[The nurse] was able to kind of say things like, ‘Each child is different’ and not to judge myself too harshly.” (Penny).

###### 3.2.2.1.1 Need for self‐care, health, and wellbeing

Mothers (*n* = 7) appreciated they needed to allow time for their own emotional, mental and physical needs as well as the baby's needs. Initially parents found balancing their own needs difficult, however, came to the conclusion that their child was often better supported when the parent was happy and healthy. There was an understanding (*n* = 4) that a child required a happy parent to thrive. “It took me a while to realize that I needed to do what was best for me for it to be best for her… I recognize when I need help, and I'm not afraid to ask for it now.” (Tiffany).

Some mothers recognized that they would benefit from placing a higher degree of emphasis on their own needs, however, found it hard to put this into practice. “I've not had time to exercise and to spend any time on myself… and I've completely run myself into the ground.” (Vanessa).

Four mothers in particular recognized that at times they benefited from being provided mental health assistance from professionals. Mothers accessed mental health supports from family members, friends, and their doctor after it was recognized by the *right@home* nurse. One mother commented on how she thought she would have suffered more if she had not been supported by the nurse. “I reckon I would have sunk into my depression really, really bad, and things would have been very, very different.” (Tiffany).

### 3.3 Theme 3. Responding to needs

All parents considered that responding to needs was an important theme. This theme consisted of their capacity to modify behavior, be open and flexible to change, acting in a child centric manner, and having confidence in their parenting ability. As previously described, there is some overlap in the recognition of needs and the response to them.

#### 3.3.1 Modification of behavior

Following on from the previous themes of recognizing needs, mothers then responded to these needs by modifying their behavior. For a change of parental behavior to be in line with responsive parenting, the modified behavioral response must be appropriate to the situation and linked to the child's previous action. At times this was difficult for mothers, however, they became better at reading their child with time, and in turn better at responding to their needs. “You hear your baby cry, and you know, you just have to go straight away. But as they get a little bit older you get to identify the different types of crying.” (Vanessa).

One mother commented on how her parenting changed after being taught by the nurses the “circle of security” (Hoffman et al., 2006), which promotes attachment and teaches the concept of the mother as a secure base. This gave her an understanding of how connecting with her child assists child development, and led to a modification of how she interacted with her child. “Instead of saying, ‘He's looking at me. Here's a toy’… I was able to say, ‘he's exploring the world’ or, ‘he's coming back in, he really needs connection with me’.” (Penny).

#### 3.3.2 Open to change and flexibility

Mothers (*n* = 12) who were open to trying new things and accepting assistance and advice from others felt they were better able to respond to their child in a more flexible manner. This allowed them to be receptive to input from others, and to try different parenting techniques. Mothers were able to then decide whether the new parenting advice was applicable to their family situation, which resulted in more harmonious family functioning. “Not all the advice is perfect for everyone. But at least something that you can try and see whether they can get things changed.” (Mei).

Mothers (*n* = 9) appreciated when advice was given in a non‐judgmental manner. This allowed the mother to be open to others’ suggestions, and able to evaluate them without feeling criticized. This openness to parenting flexibility assisted the mothers in feeling able to ask for help and implement the suggested behaviors. “The way it was also, the advice was delivered it was never a right or wrong kind of answer. It was, well here are some other types of things to try. See what works best. I thought that was really good.” (Vanessa).

#### 3.3.3 Acting in a child centric manner

Parents (*n* = 12) recognized that the child's needs were central to their parenting decision making processes. Several mothers (*n* = 5) recognized that their child benefited from a regular routine. They structured their day to reflect this, understanding that for some children deviating from this routine was difficult. One mother commented that although keeping to a routine impacted on her social life, it was worth it for the benefit it brought to her child. “If it means she is getting 12 h of sleep and she is happy the next day we are happy to do that.” (Bella).

On the whole, parents were prepared to reduce the amount of time they devoted to themselves if it was beneficial for their child. This was further evident in the context of working mothers. One mother spoke of how she had very little time to spend with her two children as she had gone back to full‐time work. She explained how she placed value on spending time with her children, despite feeling tired and having a number of conflicting demands. After work, when she has collected her children from childcare, she *“*pretty much concentrate[s] on spending time with my girls until they go to bed and after they're asleep then I do other stuff.” (Tara).

A significant element of parenting in a child centric way is to consider the impact of child's individuality when making parenting decisions. As one mother stated, “it's really important to parent the child, not to have solid views that need to be the same for every child.” (Vanessa). Another mother emphasized that her learning experience of parenting constantly changed as her children developed and grew. She gave the example of parenting her four children involved always learning new skills, as she recognized their individuality and growth. “We will always continue to learn because we deal with a 2‐year‐old, then we deal with a 10‐year‐old, but then we've still got to reach ‘what's a teenager like?’” (Melissa).

#### 3.3.4 Confidence in parenting ability

For participants, confidence in parenting ability related to the mother's belief that they can parent successfully. It involved having confidence that they are capable of making the best decisions for their child. This self‐confidence led the participants to feel they were more sensitive in their parenting, knowing they were capable of changing their responses based on their recognition of what their child requires. Confident parents were able to ask for and accept help without feeling diminished. Further, their strong confidence in their parenting ability allowed them to evaluate the usefulness of this advice to their child. “You'll get plenty of people who will put in their two cents and say, ‘you should be doing this, you should be doing that.’ But you've just got to listen to your child and listen to your own beliefs and parent that way.” (Penny).

Reassurance from the *right@home* nurse validated mothers’ parenting abilities (*n* = 12), making them more confident in their decision making. For some mothers (*n* = 2), this was the only reassurance available to them, as they were hearing negative responses from their own mother or extended family. Having a trusted outside professional endorse their mothering abilities allowed a shift in perception in the success of their mothering. “[The nurse] was always saying that I was doing a good job and to keep it up. I was like, ‘okay. I was good.’ It made me feel better.” (Jennifer).

One mother was grateful for the honesty of the nurses in teaching her how to implement new parenting approaches that were more appropriate for herself and her baby. Although the mother felt initially uncomfortable being shown a different way of parenting, she ultimately appreciated the nurse's input and felt more confident in her future parenting interactions. “[The nurses] would be honest with me, and tell me that something I was doing wasn't the right thing. And I would feel a bit like a bad mum. And they would tell me the right thing to do, and then I'd feel better because I'd know that I was doing the right thing.” (Laura).

### 3.4 Theme 4. Motivators

The theme of motivators was identified by mothers as encouraging responsivity. These motivators assisted in focusing the mothers’ actions in order to achieve positive parenting outcomes for both their children and themselves. There were three sub‐themes: child outcomes, nurse influence, and support systems.

#### 3.4.1 Child outcomes

Parents wanted the best for their child and reported that they would act in a manner that they believed will assist their child to achieve positive outcomes. Eleven mothers described how their behavior impacted on their child's outcomes, and this recognition acted as a motivator for responsive parenting.

Mothers wanted the best possible developmental outcomes for their child (*n* = 9). They identified that fulfilling physical needs and giving material possessions was not enough. They recognized that their child required the emotional support provided by love (*n* = 5), play (*n* = 6), and reassurance (*n* = 4) to fully actualize their potential. One of these mothers acknowledged the importance of interactions between parent and child in assisting their learning and development, explaining that: “You can go out and buy all the fandangle toys and everything, but if you're not reading to your child, they're not developing properly. I can understand how a lot of families might think that if they've got certain things, their child's going to be fine, but you do need to do the interaction.” (Tiffany).

#### 3.4.2 Nurse influence

Participants reported that the nurse was able to provide knowledge and support to reassure them that they were parenting appropriately (*n* = 12). This gave mothers confidence in their parenting abilities and provided a non‐judgmental space to receive advice and ask questions, motivating them to be responsive. All mothers believed the nurse home visiting intervention was beneficial in creating parenting confidence, and should be offered to mothers in the future.

One mother explained how the nurse helped her feel that her role as a mother was appreciated, giving her confidence in her capacity to be responsive. She emphasized that the nurse being in her home provided better insight into her family situation and more relevant advice. This notion of more relevant advice was mentioned by eleven women. “It makes your role as a mum feel like it's a bit more appreciated and that knowing that you're doing the right thing …When there's someone sitting right there in your home, that can see it all in front of them, and is able to give you better information and support to know that you're doing everything right.” (Michelle).

Four mothers commented on how they were happy to be able to teach others around them information that they had learnt from the nurse. “Due to the nurse, I was able to teach—or share—some of my information with others.” (Melissa). This empowered mothers by giving them knowledge they could trust, and increasing confidence and surety in their parenting skills.

Mental health difficulties were mentioned by four mothers, with two mothers specifically referencing post‐natal depression. One mother explained that she felt the nurse was supportive of her as she understood the challenges motherhood places on mental health. “It makes a big difference with just the mental health and just this feeling of support, and the reassurance too… They understand the other issues or complexities that are going on. She understood and she listened and she wasn't dismissive at all.” (Tara).

Another mother commented how she appreciated having someone who could recognize symptoms of when things were going wrong. “I had post‐natal depression. And she sent like, advised me to go to my GP. And it was really good. I wouldn't have noticed the problem or anything if they didn't point it out to me. It's like having a second mother really.” (Michelle).

#### 3.4.3 Support systems

Support systems assisted mothers to parent with autonomy, confidence, and care. Mothers identified a range of supports that helped with parenting, including their partners (*n* = 10), extended family (*n* = 11), other parents (*n* = 9), friends (*n* = 2), and professional services (*n* = 10). Decisions to increase support systems were made to allow the mother to have more capacity to give her time, skills, and knowledge to both her child and her own self‐care.

Nine women spoke about the child's father being a support, feeling that their partner assisted by co‐parenting and having consistent parenting decisions. One of these mothers commented on how she and her husband would review their parenting practice to ensure it was appropriate for their child and their situation. “My husband and I, like each week we kind of say, ‘Is there something we need to change? Is there something we need to do differently to encourage better behavior or encourage him to be more outgoing?’ (Penny)

Many mothers sought assistance from supports that were not their partner. One mother had an experience of domestic violence and made the decision to parent on her own and leave her partner. She found having the absence of a supportive partner difficult, as it left her with the sole responsibility of juggling full‐time motherhood with work and study. In particular, she found not having the emotional support of a partner especially hard. “I've only got one friend that really understands what it's like being a single parent like me, because her partner is in prison. Even she said, ‘At least I've got that emotional support, and I get the phone calls, and he helps out in that way,’ but I don't have that.” (Tiffany). She found that her emotional support came from her friends, who would “come round and visit”, “recognize triggers”, and “see if I needed any help.” (Tiffany).

Extended family was supportive for eleven mothers, with many mothers’ own parents providing physical or emotional support. An assistive co‐parenting role was often assumed by the mother or mother‐in‐law of the participant, particularly in the time following the birth of the child. “My parents helped me and everything when I got home, so, and helped me catch up on all the sleep that I'd missed.” (Laura). All mothers benefited from external supports that allowed them to focus on responsive parenting.

### 3.5 Model of responsivity

**FIGURE 1:**
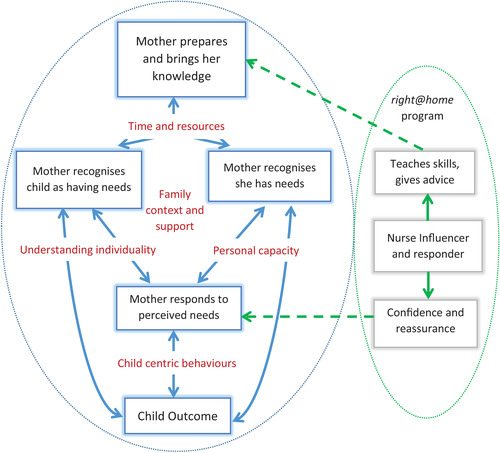
Model summarizing the process of responsive parenting for the *right@home* participants.

A model was developed to visually assist in explaining the connections between the core and sub‐themes. Figure 1 presents the model and highlights the processes described by the mothers in developing responsivity in their parenting. The model acknowledges that the interviews and experiences occur in the context of the participants’ environment, culture, past experiences, and support systems. Movement through the model can be applied to both the larger process of responsive parenting, as well as individual acts of parental responsivity.

Preparation for parenting was highlighted as the initial stage, preceding the mother's ability to recognize that both she and her child have needs that must be addressed. This preparation was augmented by the *right@home* nurse teaching skills and giving advice, both influencing and responding to the needs, knowledge, and context of the mother. The preparation and knowledge the mother brings to her parenting provides the time and resources to recognize her own and her child's needs.

Subsequent to this, the mother responded to these perceived needs, and these responses fed into child outcomes. Key to positively achieving this pathway were the mother's personal capacity to recognize and respond to her own needs, and her understanding the individuality of her child and their needs. In addition, the nurse provided reassurance and supported the mother's confidence to respond. Finally, child‐centric behaviors in responding to needs resulted in positive child outcomes.

Although the model is depicted as linear, it is dynamic in action, with the stages being both bidirectional and cyclic. Each stage has the capacity to be influenced by previous or subsequent stages in either a facilitating or inhibitory manner. For instance, when the mother displays highly sensitive responsivity she is well prepared for parenting, recognizing the needs of herself and her child, and responding appropriately. Conversely, a mother with low preparation is more likely to have more difficulty accurately recognizing needs and her responses would be less attuned to her child. In addition, a child‐centric response to needs and positive child outcomes reinforced understandings of the child's individuality and mothers’ belief in their capacity to identify (and meet) their and their child's need. The reassurance and confidence building by the *right@home* nurses supported this cyclic process.

## 4 DISCUSSION

The purpose of the current study was to explore how mothers who took part in a sustained nurse home visiting intervention understood responsive parenting, and how they viewed the intervention as an influence on their own responsive parenting. Specifically, this study aimed to investigate the influence of the *right@home* intervention on mothers’ (1) ability to actively connect with their child, (2) capacity to read child needs, and (3) perceptions of their ability to provide responsive parenting. A model describing the way responsivity was developed and supported was constructed from participants’ narrative descriptions, considering the context of the *right@home* intervention, and understanding the process of implementation and its consequences (Mason & Barnes, 2007). The four themes extracted (preparation for parenting, recognition of needs, responding to needs, and motivators) were not mutually exclusive, allowing for research questions to be addressed thematically.

It appears that, for the participants, this responsive process was not spontaneous or purely instinctive. Rather, the acquisition of responsive parenting was a product of the dynamic interaction between a woman's natural propensities and her learnt experience, which resulted in motivated, practiced, and reinforced behaviors. This is consistent with the literature that has examined mothering as a combination of a natural process and a significant learning process that increases and refines parenting skills (Kurth et al., 2014; Young et al., 2019).

This study found that mothers bring their knowledge and preparation to their parenting role. The findings highlight the importance of preparation for parenting beginning in the antenatal period, and the importance of the role of the *right@home* program nurse in supporting this preparation. This construct of maternal preparation is not often the focus of theoretical descriptions of attachment or responsive parenting. Regardless, there is evidence that the attachment process begins during pregnancy, and continues to develop over time (Trombetta et al., 2021). Parenting preparation has been discussed in the literature as a component of successful parenting. Kemp et al. (2013) highlighted how increased antenatal levels of knowledge and preparation assist in the transition to motherhood. In line with this, the current study found that psychological and behavioral preparation was an important element to the process of responsive parenting.

Consistent with theory, the current study supported the hypothesis that recognizing and responding to needs was an important part of responsive parenting. Paris et al. (2011) examined a home‐based parenting intervention with mothers experiencing post‐natal depression. They found that mothers with post‐natal depression had lowered capacity for reflective functioning, which is needed to comprehend their own thoughts and emotions, as well as those of their child. The mothers in the current study were prioritized for their experience of adversity during pregnancy, which is also associated with reduced capacity for reflective functioning (Moore et al., 2013). The *right@home* intervention was experienced by the participating women to enhance their abilities to be responsive. The program created the time and connection to resources and increased women's capacities to reflect on their own behavior, as well as their child's. This enabled women to identify and respond to both theirs and their children's unique needs. Newton et al. (2022) noted that “Helping the mother to understand the infant's internal world, needs, and unique perspective” was an effective characteristic of interventions that improved mother‐infant relationships. Importantly, in our study, there were no reports of tension between the meeting of mothers’ and children's needs. Rather, there was recognition that meeting both was needed to achieve positive child outcomes.

Women identified motivators as necessary for responsive parenting, specifically for strengthening parenting skills, promoting development of the child, and consolidating the bond between parent and child. Parenting to achieve positive child outcomes was one of the most salient themes motivating mothers’ responsive parenting. This observation is like that made by Goulet et al. (1998), who described bonding as a directional interactional process that lead to maternal attachment, providing motivation to succeed in the mothering role.

A further motivator for responsive parenting was the encouragement of the home visiting nurse and other social and professional support systems. Results suggest that the capacity of the parent to nurture is positively correlated with level of support available. Mothers stated that having their parenting abilities validated by the nurse helped them feel more confident and capable, increasing their self‐esteem and self‐efficacy. This is consistent with earlier findings from Kemp et al. (2013), whereby psychosocial support from the nurse assisted in the growth and development of mothers’ parenting knowledge, capabilities, and self‐image.

### 4.1 Strengths and limitations

The current study contributes to the body of research into early interventions and parenting with populations experiencing adversity. The current study utilized a qualitative design to collect data and give insight into the processes of development of responsive parenting. Inductive thematic analysis is a stringent and valid qualitative method, creating in‐depth and detailed exploration of responsive parenting within the *right@home* cohort. The semi‐structured interview design allowed participants to guide the direction of research by speaking about their parenting practice and what they valued about the intervention. The findings were reflective of the mother's experience, and not pre‐determined by theoretical categories.

The timing of interviews at 6 months after the conclusion of the intervention may have resulted in some recall bias regarding the direct impact of the interventions. However, this timing allowed mothers time to reflect on the program's teachings. It also gave women the opportunity to parent without the direct influence of the nurse, indicating that the responsivity described in the interviews is likely to have been consolidated into their parenting style. The small sample size and design of the study, while appropriate for exploratory analysis of this population, does not allow for generalizability to wider populations without further research. The possibility of social desirability bias must be considered, as this study relied on mothers’ report of their experiences of parenting.

## 5 CONCLUSIONS

Overall, how participants in the current study understood responsive parenting matched the theoretical definitions of parental responsivity described in the existing literature. This study, however, identified the processes that supported the development of responsivity, and nurses’ role in supporting those processes. Parents valued the intervention and the nurse input, finding the adjustment to parenting challenging, and benefitting from support and validation. Further, mothers, their children, and their broader network identified multiple social, emotional, and physical benefits from their participation in the intervention. Like previous research on parental responsivity, the mothers receiving the *right@home* intervention identified reading and responding to their child's cues as important. In the current study, mothers also said the intervention gave them the opportunity to practice and reinforce these skills. Further, mothers’ experiences included preparation for responsive parenting, while also keeping in mind the purpose of their actions as assisting their ability to parent skillfully and confidently. The complementary nature of meeting parent and child needs was highlighted. This study adds to growing literature showing that interventions aimed at assisting parenting and the parent‐child relationship are of value to parents, especially when their own support system is not strong or available.

### 5.1 Implications for practice and further research

In our study, parents stated that they learned skills that are essential to responsive parenting. These skills were enabled through three main mechanisms: attention paid to behavioral skills and the actions needed to parent (rather than vague or unclear information); promoting the parent‐child relationship; and validating the mother's experience (Paris et al., 2011). The importance of preparation for parenting in this study suggests that interventions must commence in pregnancy and have long‐term engagement with families. This is especially true for women who are experiencing socioeconomic or psychosocial adversity and may have fewer opportunities or capacity for responsive parenting. Developing and practicing the skills needed for responsive parenting does not occur quickly. They require a commitment from the mother and nurse, and other social and professional supports over time. This study also found that the meeting of mothers’ own needs was critical to responsive parenting, and was not in conflict with meeting the child's needs. Interventions to improve maternal responsivity should thus dually focus on parent and child needs, affirming mothers’ self‐care strategies. Overall, this study provides further evidence that a systematic and sustained intervention with high intensity of supports is beneficial for mothers’ understanding of and ability to provide responsive parenting. Future studies may usefully examine the model developed here and whether interventions supporting the model processes described in the model create demonstrable and quantifiable changes to responsive parenting.

## CONFLICT OF INTEREST STATEMENT

The authors have no conflicts of interest relevant to this article to disclose.

## Data Availability

We invite researchers to request access to the data from the Translational Research and Social Innovation (TReSI) Group at Western Sydney University (tresi@westernsydney.edu.au).
